# Silent existence of eosinopenia in sepsis: a systematic review and meta-analysis

**DOI:** 10.1186/s12879-021-06150-3

**Published:** 2021-05-24

**Authors:** Yao Lin, Jiabing Rong, Zhaocai Zhang

**Affiliations:** 1 Department of Critical Care Medicine, Second Affiliated Hospital, Zhejiang University School of Medicine, Hangzhou, China; 2 Department of Cardiology, Cardiovascular Key Laboratory of Zhejiang Province, Second Affiliated Hospital, Zhejiang University School of Medicine, Hangzhou, China

**Keywords:** Sepsis, Diagnosis, Eosinopenia, Biomarker, Meta-analysis

## Abstract

**Background:**

Sepsis is a life-threatening and time-critical medical emergency; therefore, the early diagnosis of sepsis is essential to timely treatment and favorable outcomes for patients susceptible to sepsis. Eosinopenia has been identified as a potential biomarker of sepsis in the past decade. However, its clinical application progress is slow and its recognition is low. Recent studies have again focused on the potential association between Eosinopenia and severe infections. This study analyzed the efficacy of Eosinopenia as a biomarker for diagnosis of sepsis and its correlation with pathophysiology of sepsis.

**Method:**

The protocol for this meta-analysis is available in PROSPERO (CRD42020197664). We searched PubMed, EMBASE, Web of Science, and Cochrane Central Register of Controlled Trials CENTRAL databases to identify studies that met the inclusion criteria. Two authors performed data extraction independently. The pooled outcomes were calculated by TP (true positive), FP (false positive), FN (false negative), TN (true negative) by using bivariate meta-analysis model in STATA 14.0 software. Meanwhile, possible mechanisms of sepsis induced Eosinopenia was also analyzed.

**Results:**

Seven studies were included in the present study with a total number of 3842 subjects. The incidence of Eosinopenia based on the enrolled studies varied from 23.2 to 92.7%. For diagnosis of sepsis, the pooled sensitivity, specificity, positive likelihood ratio, negative likelihood ratio and diagnostic odds ratio of Eosinopenia were 0.66 (95%CI [0.53–0.77]), 0.68 (95%CI [0.56–0.79]), 2.09 (95%CI [1.44–3.02]), 0.49 (95%CI [0.34–0.71]) and 4.23 (95%CI [2.15–8.31]), respectively. The area under the summary receiver operator characteristic curve (SROC) was 0.73 (95%CI [0.68–0.76]). Meta-regression analysis revealed that no single parameter accounted for the heterogeneity of pooled outcomes. For each subgroup of different eosinopenia cutoff values (50, 40, ≤25, 100), the sensitivity was 0.61, 0.79, 0.57, 0.54, and the specificity was 0.61, 0.75, 0.83, 0.51, respectively.

**Conclusions:**

Our findings suggested that Eosinopenia has a high incidence in sepsis but has no superiority in comparison with conventional biomarkers for diagnosis of sepsis. However, eosinopenia can still be used in clinical diagnosis for sepsis as a simple, convenient, fast and inexpensive biomarker. Therefore, further large clinical trials are still needed to re-evaluate eosinopenia as a biomarker of sepsis.

**Supplementary Information:**

The online version contains supplementary material available at 10.1186/s12879-021-06150-3.

## Background

As “a life-threatening organ dysfunction caused by a dysregulated host response to infection” [1], sepsis has been recognized as a major threat to global health causing millions of deaths per year globally according to WHO, and a true time-critical medical emergency susceptible to death [2]. Therefore, an accurate and quick diagnostic testing is essential to improve outcomes in patients with sepsis. Although working as the gold standard for identifying infectious conditions, microbial culture has obvious limitations such as time-consuming delay and considerable false negative results [3]. Thus, seeking timely, sensitive and specific biomarkers has become the research focus in this field, with C reaction protein (CRP), procalcitonin (PCT) and white blood cell count as potential candidates, while the efficiency is far below satisfaction [3, 4].

Eosinophils, comprising 1–3% of circulating leukocytes, play a role in host defense against helminths and the propagation of allergic conditions. Eosinophils are considered as an integral part of immune and inflammatory network, and homeostatic regulation as well in recent years [5], all of which play an indispensable role in the pathophysiology of sepsis. The reduction of circulating eosinophil count (namely eosinopenia) in response to acute infection was firstly described in 1893 by Zappert et al. [6], and is now acknowledged as a signal of acute infection [7, 8]. There are many emerging studies focusing on the correlation between eosinopenia and bloodstream infection or severe infection in intensive care unit recent years, which actively explored the potential diagnostic and prognostic value of eosinopenia [9–13], or the capacity to guide antimicrobial therapy [14].

The feasibility of eosinopenia in diagnosis of sepsis was firstly tested by Abidi et al., who showed that eosinopenia had good sensitivity and specificity in diagnosing sepsis [15], and subsequent study of Shaaban and his colleagues also demonstrated the great diagnostic value of eosinopenia for detecting sepsis [16]. Given that there was a significant correlation between sepsis and eosinopenia, eosinopenia used to arouse much attention and be considered as a promising marker of sepsis. Several studies were carried out in addressing the value of eosinopenia to diagnose sepsis recently [15–21] and to predict the prognosis of sepsis [22–24], although there were pretty much inconsistence and controversy. Moreover, since the outbreak of COVID-19 (coronavirus disease 2019), eosinopenia, which is one of the most significant features of COVID-19 [25], has become a hot topic again [26], because of its potential diagnostic and prognostic value [27–30].

With emerging studies on eosinopenia as a marker of severe infection and sepsis, we determine to perform the present meta-analysis to reevaluate whether eosinopenia is a good biomarker for diagnosis of sepsis.

## Methods

### Study design and literature search

The present meta-analysis was performed according to the Preferred Reporting Items for Systematic Reviews and Meta-Analysis (PRISMA) statement [31]. The protocol for this meta-analysis is available in PROSPERO (CRD42020197664). Studies that investigated the diagnostic efficacy of eosinopenia for sepsis were searched for inclusion. Discrepancies were resolved by the consensus of all authors.

A comprehensive electronic search of the PubMed, EMBASE, Web of Science, and Cochrane Central Register of Controlled Trials CENTRAL databases was performed. Then we reviewed the reference lists of included articles to obtain additional relevant articles. No language restriction or publication date restrictions were applied, and the date of our search was until 9 July 2020.

We used the keywords “sepsis” and “eosinopenia” to search articles, and the process of study selection is schematically presented in the PRISMA flow diagram. The detailed search strategy is showed as in Suppl. Table 4.

### Selection criteria

Studies were selected based on the following inclusion criteria: (1) enough data to calculate the outcome data (true positive (TP), false positive (FP), false negative (FN), true negative (TN)); (2) the gold standard for diagnosis of sepsis was defined in the study; (3) prospective or retrospective study design; (4) only adult patients were involved. The literatures were excluded if they are (1) reviews, case reports, editorials and animal experiments; (2) only healthy people were used as controls.

### Data extraction

Two authors (LY, RJB) independently collected data referring to study and patient characteristics. A third author (ZZC) independently assessed these data in case of inter-reviewer discrepancies. The extracted information from each study included first author name, year of publication, country, study design, sample size, control patients, cutoff value, prevalence, male, mean age, timing of eosinophils counts assessment, reference standard and outcome data (TP, FP, FN, TN). For studies providing multiple eosinopenia cutoff values, the outcome data of all cutoff values were extracted.

### Quality assessment

The methodological quality of the included studies was tested with the QUADAS-2 tool by two authors. QUADAS-2 consists of four sections: patient selection, index text, reference standard, and flow and timing [32]. The included studies were ranked as low risk, high risk, or unclear risk. We performed the quality assessment using Review Manager (RevMan), version 5.3 (Cochrane Collaboration).

### Statistical analysis

The pooled sensitivity, specificity, positive likelihood ratio (PLR), negative likelihood ratio (NLR), diagnostic odds ratio (DOR), AUC, and corresponding 95% credible interval (CI) were calculated by TP, FP, FN, TN using a bivariate regression model using STATA 14.0 software. Deek’s funnel plot was used to detect publication bias, with *P* < 0.05 indicating publication bias. A Fagan plot was assembled for the visual presentation of diagnostic performance.

We used Spearman correlation coefficient to detect threshold effects by using Meta-DiSc software (version 1.4), and *P* value< 0.05 indicates a significant threshold effect. We used I^2^ to describe the heterogeneity in the meta-analysis. I^2^ value≥50% is considered as a significant heterogeneity. Then, the possible sources of heterogeneity were explored by conducting a meta-regression analysis and sensitivity analysis. The examined parameters in meta-regression included cutoff (≥50/< 50), control patient (SIRS/non-infection), sample size (≥200/< 200), type of study (prospective/retrospective), and country (Europe and America/others). A sensitivity analysis was conducted to examine the influence of each study on the meta-analysis, by calculating the pooled outcomes after omitting one study at a time. The impact of eosinopenia cutoff used in each study was further investigated by performing a subgroup analysis.

## Result

### Included studies

**Fig. 1:**
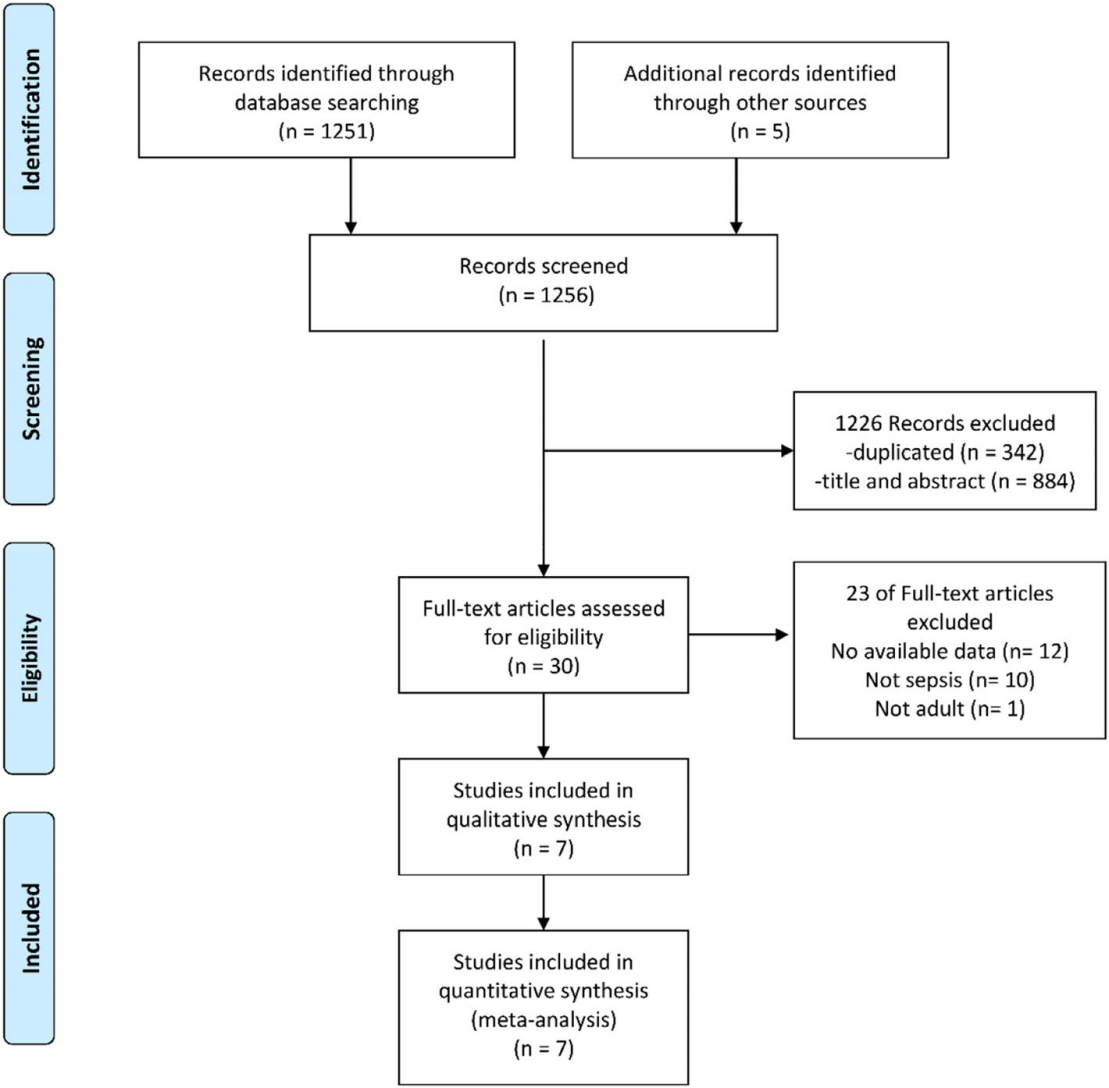
PRISMA Flow diagram

Seven studies that represented 12 trials were included in this meta-analysis (using different cutoff values in the same studies was regarded as different trials), with a total number of 3842 subjects. Among which, 1152 individuals were diagnosed as sepsis, while the rest of 2690 individuals were considered as non-sepsis. A summary of the characteristics (first author name, year of publication, country, study design, sample size, control patients, cutoff value, prevalence, male, mean age, and timing of Eosinophils counts assessment) of the 7 included studies is outlined in Table 1. The data used for the construction of the 2 × 2 table is presented in Suppl. Table 1, along with the cutoff values of each study. A flow diagram was assembled to describe the details of the study selection process (Fig. 1).

**Table 1:** The characteristics of included studies

Author	Year	Country	Study design	Clinical Setting	Reference standard	Severity of sepsis	Sample Size	Prevalence	Male	Mean/median Age	Control Patients	Cutoff (Cells/mm^3)	Timing of Eosinophils counts assessment
Abidi [15]	2008	Morocco	Prospective	ICU	ACCP/SCCM^1^	Sepsis; severe sepsis; septic shock	177	120/177	101/177	42	SIRS + Negative	50	Admission
											SIRS	40	
Anand [17]	2016	India	Prospective	ICU	ACCP/SCCM	severe sepsis	170	125/170	107/170	52	SIRS	50	Admission
Garnacho [18]	2014	Spain	Prospective	ICU	ACCP/SCCM	severe sepsis; septic shock	160	117/160	79/160	63	SIRS	25	Day1
													Day2
Moura [21]	2011	Brazil	Retrospective	ICU	ACCP/SCCM	Sepsis; severe sepsis; septic shock	282	99/282		58.6 + −20	SIRS + Negative	100	Admission
Lavoignet [19]	2016	France	Retrospective	ED	ACCP/SCCM	Sepsis; severe sepsis; septic shock	692	125/692	0.45	59 + − 17.2	Non-infection	100	Continuous testing in one-week duration
												50	
												10	
López [20]	2010	Spain	Retrospective	ICU	ACCP/SCCM	Sepsis; severe sepsis; septic shock	244	55/244	165/244	54.4 + − 19.8	Non-infection	10	Day1
												50	
												40	Day5
Shaaban [16]	2010	USA	Prospective	ICU	ACCP/SCCM	Sepsis; severe sepsis; septic shock	68	31/68	33/68	68	SIRS + noSIRS	50	Admission

Footnotes: *ACCP/SCCM*^1^ American College of Chest Physicians/Society of Critical Care Medicine; Negative: negative results of bacterial culture, *SIRS* systemic inflammatory response syndrome, *ICU* intensive care unit

### Quality assessment

**Fig. 2:**
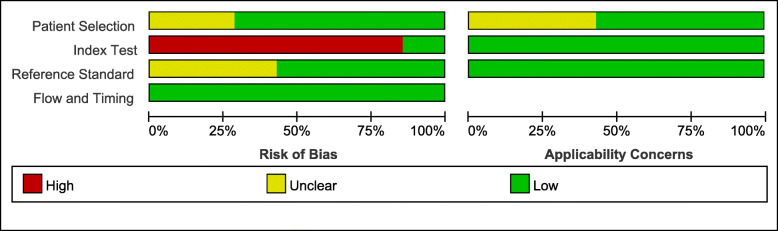
QUADAS2 for quality assessment

We used the QUADAS-2 tool to evaluate the quality of included studies, and results are illustrated in Fig. 2 and summarized in Suppl. Fig. 1. High risk of bias was indicated in the section of index test, as 5 studies did not use pre-defined cutoff values to calculate sensitivity and specificity, but rather used the optimal ones, and 1 study did not mention whether it used a pre-defined value. When considering applicability concerns, 3 studies showed potential problems in patient selection.

### Publication bias

The Deeks’ funnel plot indicated no evidence of publication bias (*P* = 0.65) (Suppl. Fig. 2).

### Outcomes

**Fig. 3:**
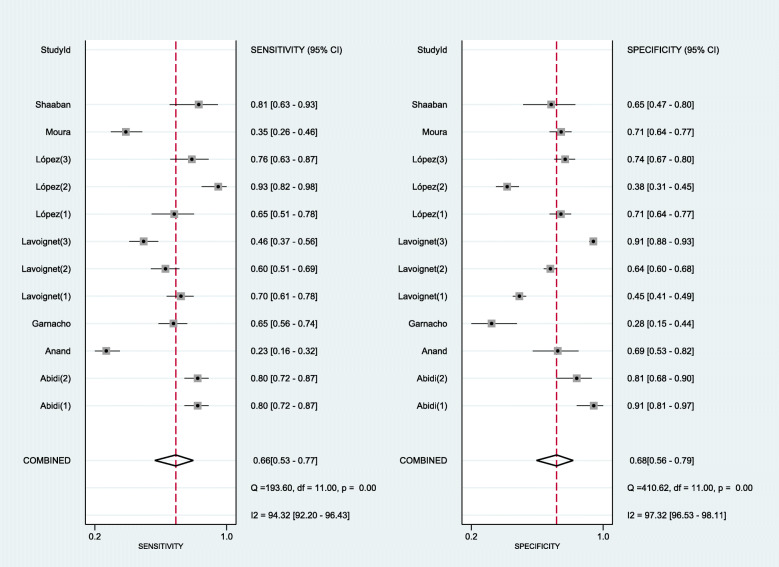
Forest plots of the pooled sensitivity and 156 specificity for eosinopenia in diagnosing sepsis

**Fig. 4:**
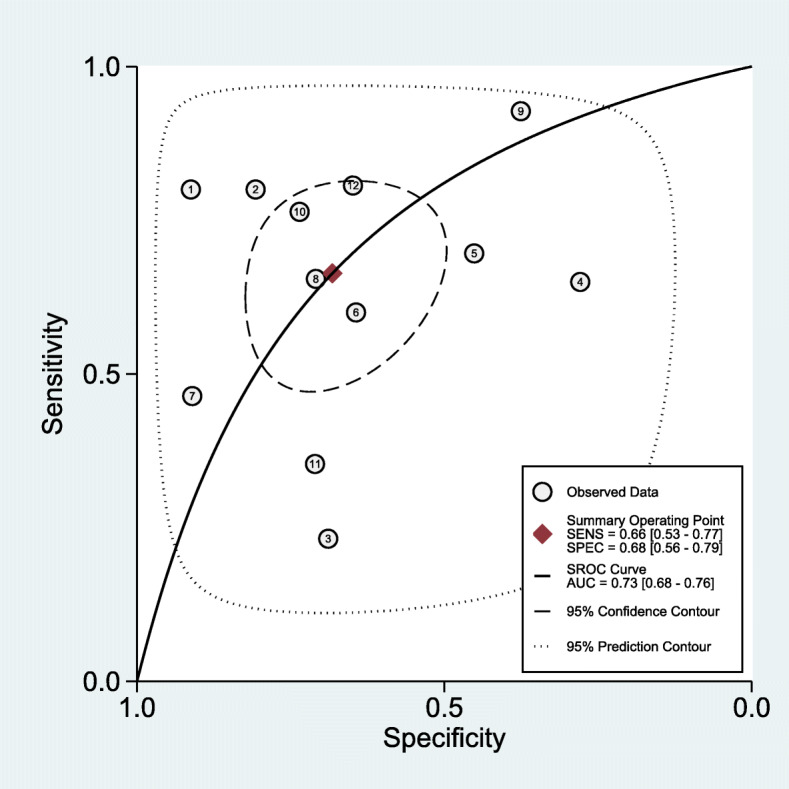
Summary receiver operator characteristic plots with 95% CI of sensitivity against specificity of eosinopenia for diagnosing sepsis

The pooled sensitivity of eosinopenia for the prediction of sepsis was 0.66 (95%CI [0.53–0.77]) and the pooled specificity was 0.68 (95%CI [0.56–0.79]) (Fig. 3). The pooled PLR was 2.09 (95%CI [1.44–3.02]) and the pooled NLR was 0.49 (95%CI [0.34–0.71]) (Suppl. Fig. 3). The diagnostic odds ratio was 4.23 (95%CI [2.15–8.31]) (Suppl. Fig. 4) and the area under the curve (AUC) was 0.73 (95%CI [0.68–0.76]) (Fig. 4). Fagan’s nomogram for likelihood ratios showed that by using eosinopenia to diagnose sepsis, the post-probability increased to 47% and decreased to 17%, when the pre-test probability was set at 30% (Suppl. Fig. 5).

The Spearman correlation coefficient (*ρ* = 0.056, *P* = 0.863) indicated that no threshold effect existed.

**Fig. 5:**
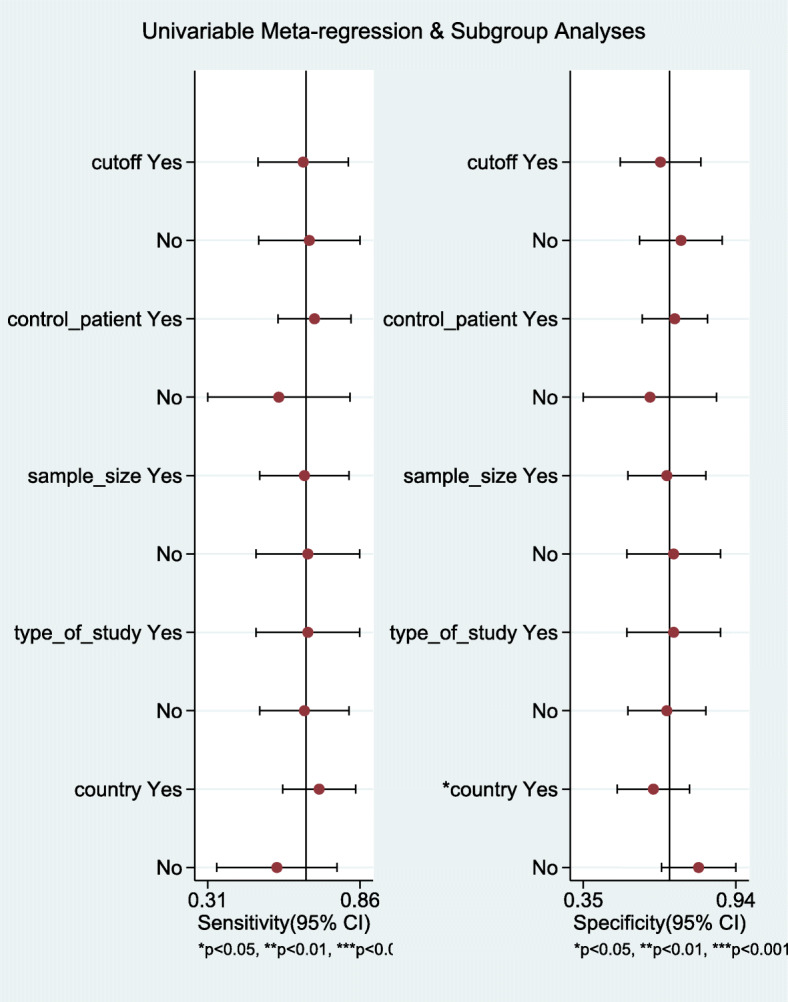
Meta-regression analysis. Legend: Yes/No represents that: cutoff (≥50/<50), control patient (SIRS/non-infection), sample size (≥200/<200), type of study (prospective/retrospective), country (Europe and America/others)

**Fig. 6:**
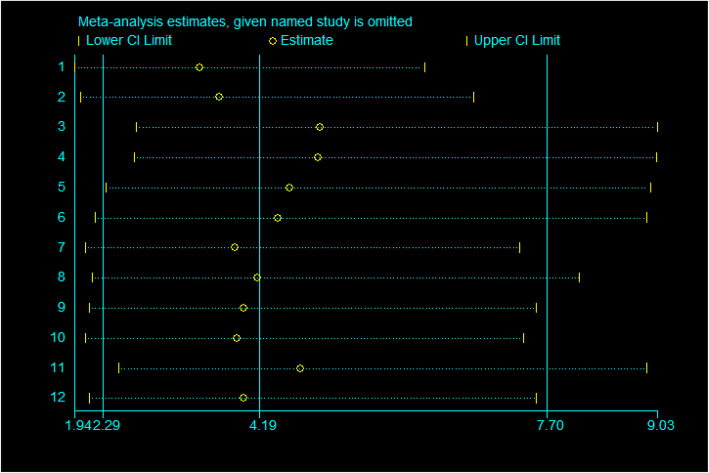
Sensitivity analysis

We used I^2^ to measure the heterogeneity in our meta-analysis. For the pooled sensitivity, specificity, PLR, NLR, DOR, the I^2^ values were 94.32, 97.32, 91.75, 92.12, 100%, which indicated a significant heterogeneity in the majority of analysis. To determine the sources of heterogeneity, we brought cutoff, control patient, sample size, study design, and country into meta-regression analysis (Fig. 5 and Suppl. Fig. 6), and the results showed that the examined covariates could not account for the heterogeneity. We also conducted a sensitivity analysis to detect the influence of each study on the pooled DOR (Fig. 6) and overall outcomes (Suppl. Table 2). This Leave-one-out analysis indicated that no single study exerted significant influence on the results of the meta-analysis.

As the cutoff values varied greatly in these included trials, we performed a cutoff subgroup analysis. The results are shown in Suppl. Table 3. For eosinopenia cutoff values (50, 40, <=25, 100), the sensitivity was 0.61, 0.79, 0.57, 0.54, and the specificity was 0.61, 0.75, 0.83, 0.51, respectively.

## Discussion

Eosinopenia is a response to acute inflammation, displaying a decreasing number of eosinophils in circulation, an accumulation of eosinophils at the inflammatory sites, and an inhibition of eosinophils production in bone marrow [33]. Eosinophils are increasingly recognized to play a critical role in modulating local and systemic immune and inflammatory responses [5]. Given that sepsis is accompanied by dysregulated immune responses and inflammatory cascades, it is reasonable to regard eosinopenia as an indication of pathophysiological status during sepsis. In the past decade, eosinopenia has aroused much attention in its diagnostic and prognostic values for bacterial infection and sepsis, with some studies showing promising results [9, 10, 16–21, 23, 24]. However, the results of currently available studies on the diagnostic performance of eosinopenia are quite different, which is manifested by a range of sensitivity from 23.2 to 92.5% and a range of specificity from 28.57 to 91% [15–21]. The diagnostic value of eosinopenia in sepsis was falling out of the limelight in recent years due to some unsatisfactory results of clinical studies, however, the incidence of eosinopenia in patients with sepsis was relatively high according to the data of our included studies (Suppl. Table 5). Eosinopenia is quite common in sepsis, which indicates that there may be some undiscovered correlations between eosinopenia and the pathophysiology of sepsis. Eosinopenia is still worthy of our further studies, especially in the situation that we are updating our knowledge of the pathophysiology of sepsis after the emergence of Sepsis 3.0 [1].

The present meta-analysis covers studies from 2008 to 2016 on the value of eosinopenia for diagnosis of sepsis in adults, which has not been performed previously. Our primary finding is that eosinopenia is only a moderate biomarker for the diagnosis of sepsis, as shown the area under the SROC curve was 0.73, the DOR was 4.23, and the pooled sensitivity and specificity was 0.66 and 0.68 respectively. Eosinopenia showed no superiority to the two most widely used biomarkers, PCT and CRP, when being used to diagnose sepsis according to a latest meta-analysis [34]. However, a clinical test for eosinopenia is more convenient, faster, and inexpensive compared to CRP and PCT. Therefore, in spite of limited diagnostic performance, eosinopenia is still a promising and competitive biomarker in clinical practice.

However, after a further examination of the literatures used for this meta-analysis, we found that there was significant statistical heterogeneity, which had a potential causal relationship with country, sample size, study designs, control setting, and cut-off values etc. Firstly, the 7 included studies were conducted in 7 different countries (suppl. Table 2) from Asia, Africa, Europe, and America, where medical levels and ethnic characteristics vary greatly. Secondly the sample size ranged from 68 to 692 as we know that inappropriate sample size may contribute to an unreliable result. However, according to the results of the sensitivity analysis, no individual study that was removed could significantly change the pooled DOR estimated by the remaining studies. Thirdly, we included all observation studies (prospective and retrospective), and it is universally acknowledged that prospective studies have a higher credibility than retrospective studies. Fourthly, some included studies assessed only SIRS patients as controls while the other studies assessed SIRS patients and infected patients without SIRS as controls or just used non-infectious patients as controls (without determining whether they had SIRS). It is more difficult to differentiate sepsis from SIRS in clinical practice because of the similar clinical signs. Fifthly, the cutoff values of eosinopenia among these included trials range from 10 to 100 (cells/mm^3^), which may also be the sources of heterogeneity, and then we performed a subgroup analysis. When using the cutoff value = 40 cells/mm^3^, eosinopenia showed the best diagnostic performance with a sensitivity of 0.79 and a specificity of 0.75, although the insufficient number of included trials in some subgroups is a big limitation of the subgroup analysis. However, no individual factors could account for the heterogeneity except the parameter of country can interpret the heterogeneity of specificity alone. Most likely there are undiscovered factors contribute to the heterogeneity. Furthermore, according to this meta-regression analysis, study design exerted no observable influence on pooled sensitivity and specificity, while selecting SIRS patients as controls exactly exhibited lower sensitivity and specificity, although there is no statistical significance.

To further explore the potential value of eosinopenia for diagnosis of sepsis in clinical practice, we constructed a Fagan nomogram, which showed that testing eosinopenia could help increase the post-probability to 47% and reduce the post-probability to 17%, with setting a pre-test probability of 30%. In summary, our results did not support eosinopenia as a clinically useful tool for diagnosis of sepsis.

### Implications for future researches

Because of the internal heterogeneity and small number of including studies, our results show only moderate association between eosinopenia and sepsis. However, the reasons why eosinopenia could not be neglected in sepsis at least include: (1) the tight correlation between eosinopenia and acute inflammation or severe infection which is still a popular and cutting-edge research area in recent years [9–14, 27–30, 2) the central role of eosinopenia in the regulation of immune network and homeostasis which have been proved as pivotal parts in the pathophysiology of sepsis [35].

Future researches are supposed to focus on the following three aspects: (1) whether eosinopenia could contribute to the diagnosis of sepsis caused by some specific pathogens; (2) whether eosinopenia show a significant advantage in the era of sepsis-3.0, given that the studies included in our meta-analysis were almost conducted before the emergence of sepsis-3.0 criteria [1, 3) The two widely studied biomarkers, PCT and CRP, and other novel biomarkers, such as neutrophil CD64, presepsin, IL-27, cfDNA and miRNAs, also have been intensely examined, showing no definite and satisfactory diagnostic efficacy individually, while combination may provide better sensitivity and specificity [4, 36].

### Limitation of the study

Firstly, the number of included studies is small, in spite of the extensive search of literature. Secondly, a significant heterogeneity exists in our analysis, and we did not find a single factor to account for it. Thirdly, these included studies spanned from 2008 to 2016, and was conducted in 7 different countries across 4 continents, which involves complicated factors such as healthcare system, detection sensitivity, antibiotic utilization, and ethnic characteristics.

## Conclusion

Collectively, our results revealed that eosinopenia is not a satisfactory but still a practical and economic biomarker of sepsis in spite of its only moderate sensitivity and specificity. This condition could be due to the insufficient number of included studies, thus more comprehensive clinical studies should be included to make a definite conclusion. Moreover, future studies can focus on the combination of several biomarkers instead of one single biomarker to diagnose sepsis.

## Supplementary Information

**Additional file 1: **Suppl. Table 1**.** The data used for the construction of the 2 x 2 table. **Suppl. Table 2.** Sensitivity analysis of the influence of each study on the overall outcomes. **Suppl. Table 3.** Subgroup analysis of cutoff values. **Suppl. Table 4.** The details of search strategy. **Suppl. Table 5.** The incidence of eosinopenia in patients with sepsis.

**Additional file 2: Suppl. Fig. 1.** Summary of QUADAS2 for quality assessment.

**Additional file 3: Suppl. Fig. 2.** Deek’s funnel plots evaluating publication bias.

**Additional file 4: Suppl. Fig. 3.** Forest plots of the pooled PLR and NLR.

**Additional file 5: Suppl. Fig. 4.** Forest plots of the pooled DOR.

**Additional file 6: Suppl. Fig. 5.** Fagan diagram.

**Additional file 7: Suppl. Fig. 6.** Summary of meta-regression analysis.

## Data Availability

All data generated during this study are included in this published article and its supplementary information files.

## Abbreviations

TP: True positive
FP: False positive
FN: False negative
TN: True negative
95%CI: 95% credible interval
PLR: Positive likelihood ratio
NLR: Negative likelihood ratio
DOR: Diagnostic odds ratio
AUC: Area under the curve
SROC: Summary receiver operator characteristic
WHO: World Health Organization
CRP: C reaction protein
PCT: Procalcitonin
SIRS: Systemic inflammatory response syndrome
ICU: Intensive care unit
cf-DNA: Cell-free plasma DNA
QUADAS-2: Quality Assessment of Diagnostic Accuracy Studies
