# Supplementary material for: Silent existence of eosinopenia in sepsis: a systematic review and meta-analysis
Source: BMC Infect Dis. 2021 May 24;21:471. doi: 10.1186/s12879-021-06150-3 (PMC8142617; doi:10.1186/s12879-021-06150-3)
Supplement: Supplementary file 1 — Additional file 1: Suppl. Table 1. The data used for the construction of the 2 x 2 table. Suppl. Table 2. Sensitivity analysis of the influence of each study on the overall outcomes. Suppl. Table 3. Subgroup analysis of cutoff values. Suppl. Table 4. The details of search strategy. Suppl. Table 5. The incidence of eosinopenia in patients with sepsis. [file 12879_2021_6150_MOESM1_ESM.zip › Suppl. table 4-search strategy.docx]

(("Eosinophils"[Mesh]) OR ((eosinophil*[Title/Abstract]) OR (eosinopenia[Title/Abstract]))) AND (("Sepsis"[Mesh]) OR (((((((((((((((septic[Title/Abstract]) OR (Pyemia[Title/Abstract])) OR (Pyemias[Title/Abstract])) OR (Pyohemia[Title/Abstract])) OR (Pyohemias[Title/Abstract])) OR (Pyaemia[Title/Abstract])) OR (Pyaemias[Title/Abstract])) OR (Septicemia[Title/Abstract])) OR (Septicemias[Title/Abstract])) OR ("Poisoning, Blood"[Title/Abstract])) OR ("Blood Poisoning"[Title/Abstract])) OR ("Blood Poisonings"[Title/Abstract])) OR ("Poisonings, Blood"[Title/Abstract])) OR ("Severe Sepsis"[Title/Abstract])) OR ("Sepsis, Severe"[Title/Abstract])))

To reduce the number of results, for searches in Embase and Web of Science, we also used the search terms “NOT (review OR letter OR editorial OR” animal experiment” OR “proceeding paper” OR “poster presentation” OR “meta-analysis” OR “case report”)”

Cochrane library

ID Search Hits

#1 MeSH descriptor: [Sepsis] explode all trees 4453

#2 (eosinopenia or eosinophil):ti,ab,kw (Word variations have been searched) 4293

#3 MeSH descriptor: [Eosinophils] explode all trees 779

#4 #2 or #3 4293

#5 (sepsis):ti,ab,kw OR (Pyemia):ti,ab,kw OR (Pyemias):ti,ab,kw OR (Pyohemia):ti,ab,kw OR (Pyohemias):ti,ab,kw 10701

#6 (Pyaemia):ti,ab,kw OR (Pyaemias):ti,ab,kw OR (Septicemia):ti,ab,kw OR (Septicemias):ti,ab,kw OR (Poisoning, Blood):ti,ab,kw 1608

#7 (Blood Poisoning):ti,ab,kw OR (Blood Poisonings):ti,ab,kw OR (Poisonings, Blood):ti,ab,kw OR (Severe Sepsis):ti,ab,kw OR (Sepsis, Severe):ti,ab,kw 3319

#8 (septic):ti,ab,kw 4344

#9 #1 or #5 or #6 or #7 or #8 15622

#10 #4 and #9 20
