# Supplementary material for: Silent existence of eosinopenia in sepsis: a systematic review and meta-analysis
Source: BMC Infect Dis. 2021 May 24;21:471. doi: 10.1186/s12879-021-06150-3 (PMC8142617; doi:10.1186/s12879-021-06150-3)
Supplement: Supplementary file 1 — Additional file 1: Suppl. Table 1. The data used for the construction of the 2 x 2 table. Suppl. Table 2. Sensitivity analysis of the influence of each study on the overall outcomes. Suppl. Table 3. Subgroup analysis of cutoff values. Suppl. Table 4. The details of search strategy. Suppl. Table 5. The incidence of eosinopenia in patients with sepsis. [file 12879_2021_6150_MOESM1_ESM.zip › Suppl. table 5-incidence of eosinopenia.docx]

Supplementary Table 5: The incidence of eosinopenia in patients with sepsis (based on different cutoffs)

| Cutoff (Cells/mm^3) | Case of eosinopenia in sepsis | Patients with sepsis | Incidence of eosinopenia in sepsis |
| --- | --- | --- | --- |
| 50 | 276 | 456 | 60.5% |
| 40 | 138 | 175 | 78.9% |
| 25 | 76 | 117 | 65.0% |
| 10 | 94 | 180 | 52.2% |
| 100 | 122 | 224 | 54.5% |
