# Supplementary figures and images for: Silent existence of eosinopenia in sepsis: a systematic review and meta-analysis
Source: BMC Infect Dis. 2021 May 24;21:471. doi: 10.1186/s12879-021-06150-3 (PMC8142617; doi:10.1186/s12879-021-06150-3)

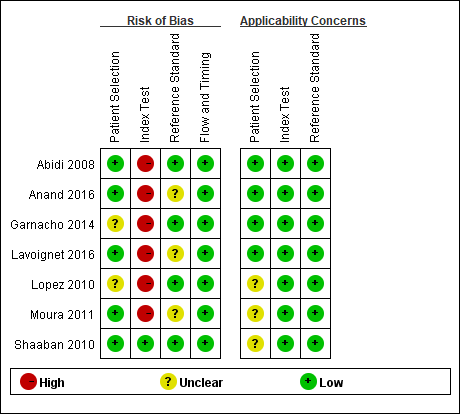

Supplement: Supplementary file 2 — Additional file 2: Suppl. Fig. 1. Summary of QUADAS2 for quality assessment. [file 12879_2021_6150_MOESM2_ESM.tif]

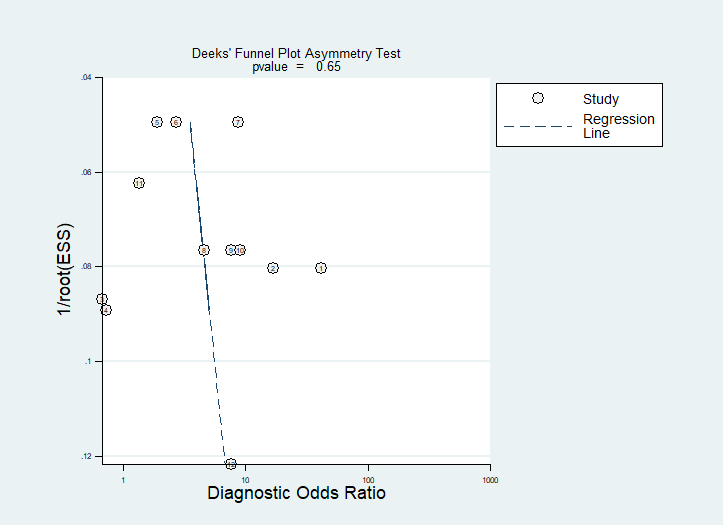

Supplement: Supplementary file 3 — Additional file 3: Suppl. Fig. 2. Deek’s funnel plots evaluating publication bias. [file 12879_2021_6150_MOESM3_ESM.tif]

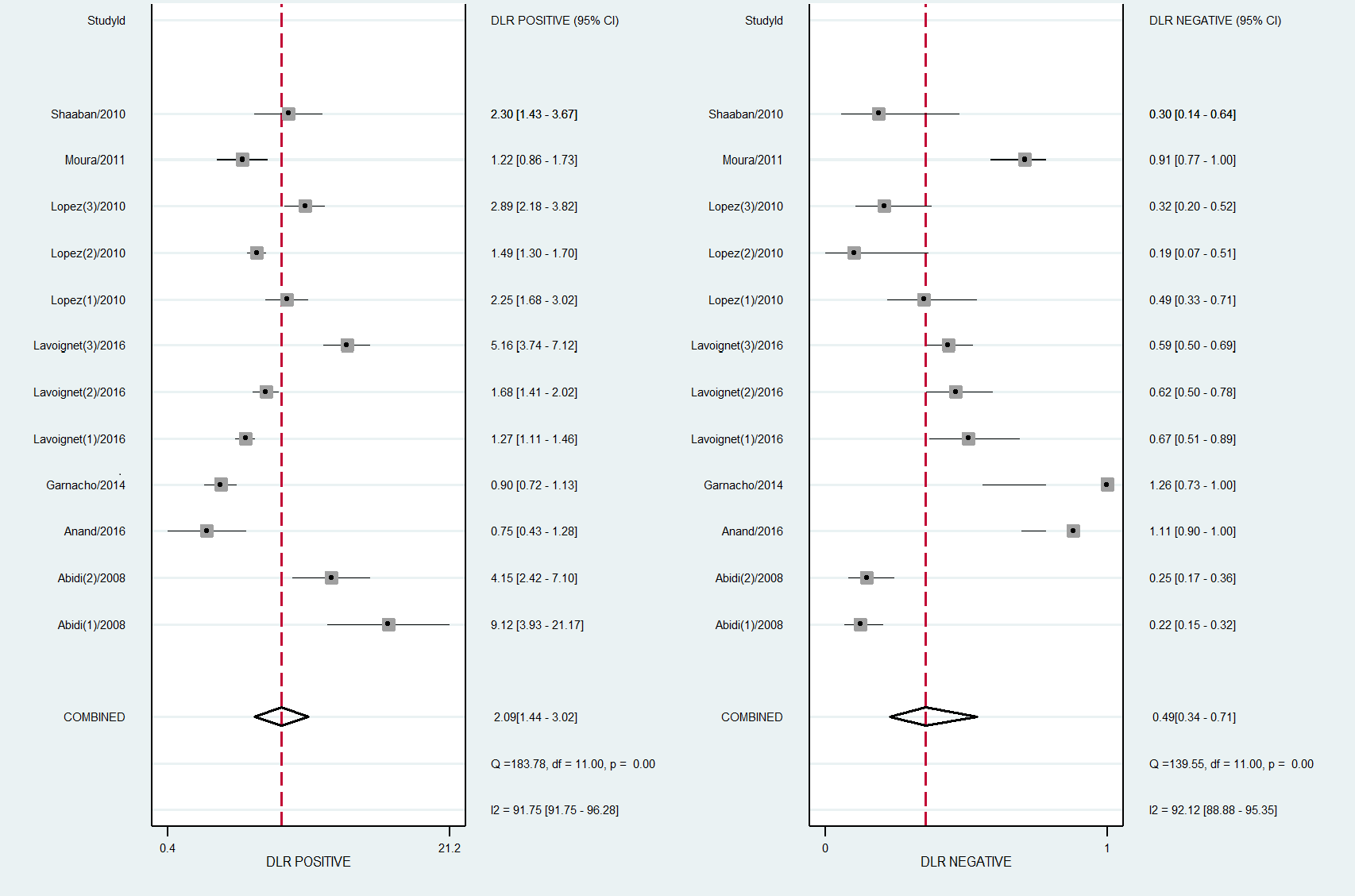

Supplement: Supplementary file 4 — Additional file 4: Suppl. Fig. 3. Forest plots of the pooled PLR and NLR. [file 12879_2021_6150_MOESM4_ESM.tif]

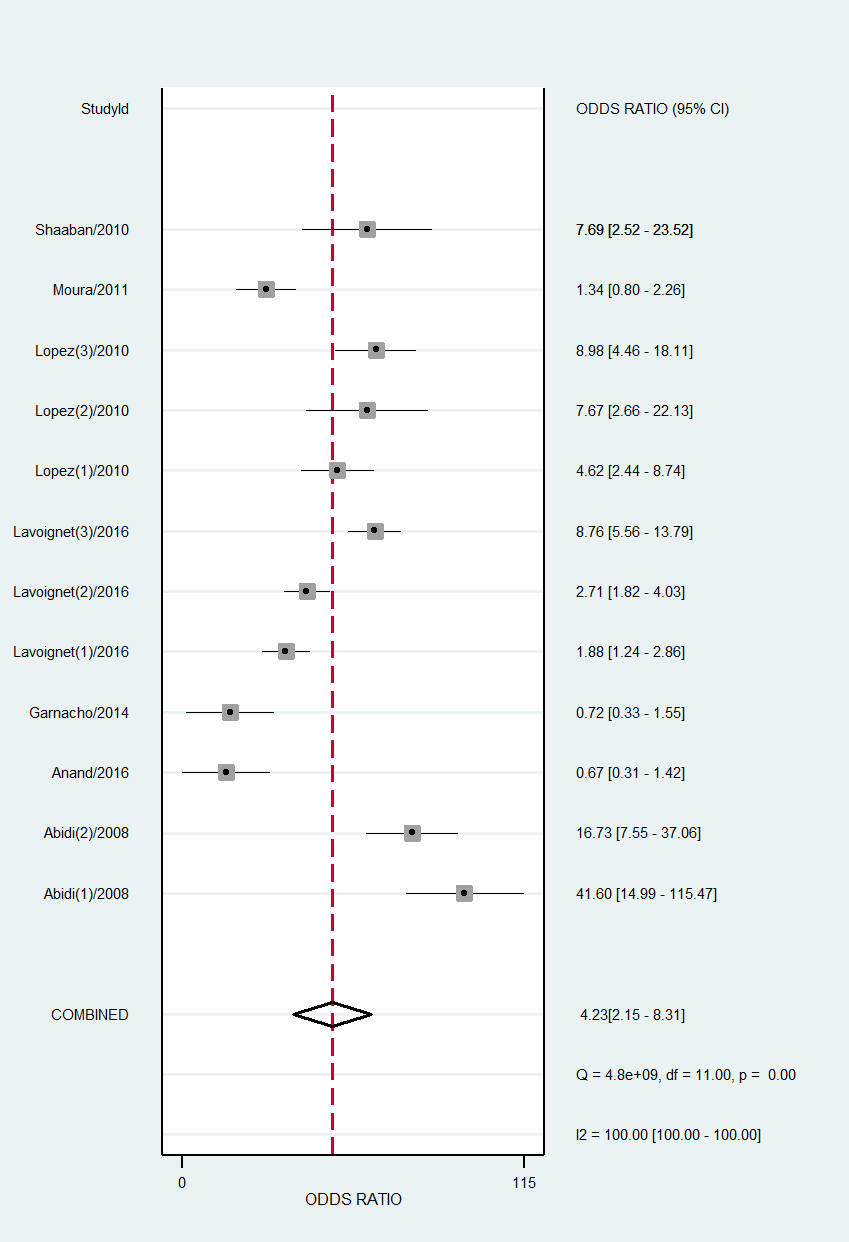

Supplement: Supplementary file 5 — Additional file 5: Suppl. Fig. 4. Forest plots of the pooled DOR. [file 12879_2021_6150_MOESM5_ESM.tif]

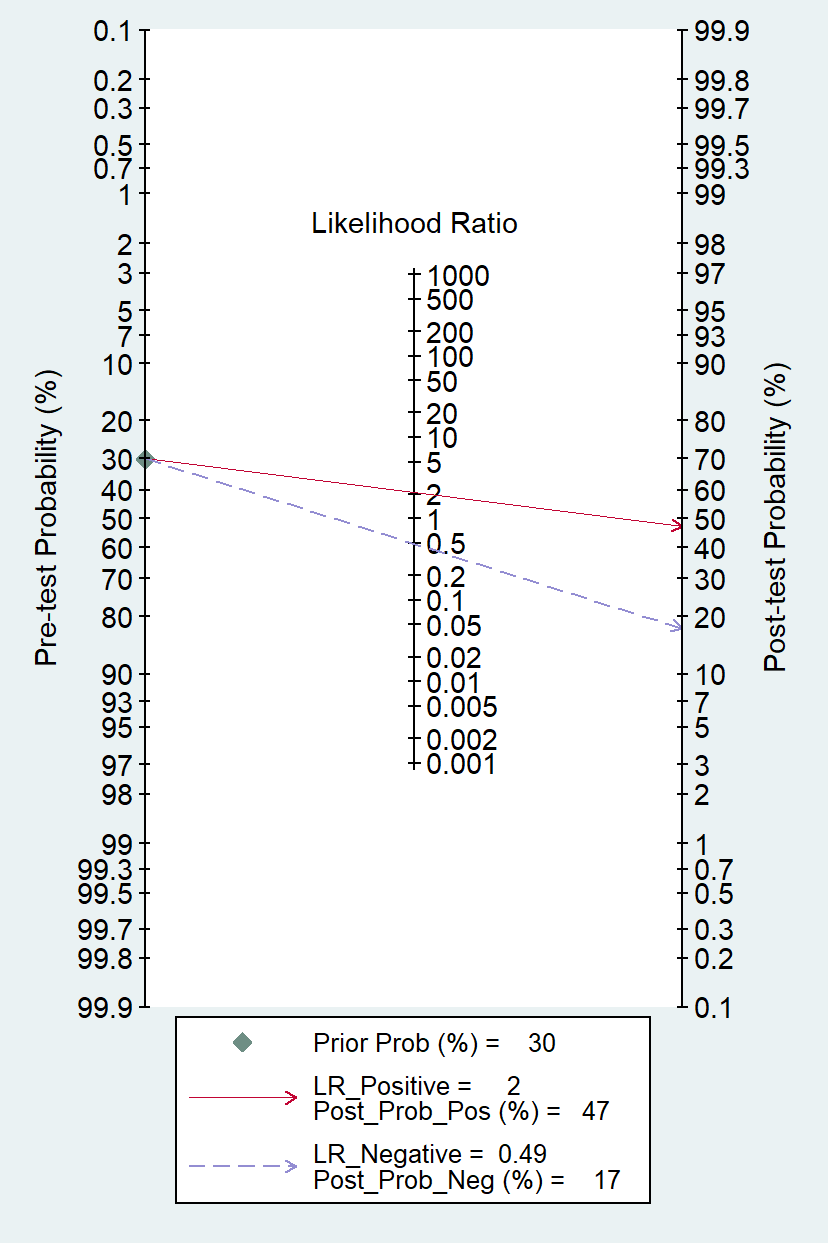

Supplement: Supplementary file 6 — Additional file 6: Suppl. Fig. 5. Fagan diagram. [file 12879_2021_6150_MOESM6_ESM.tif]

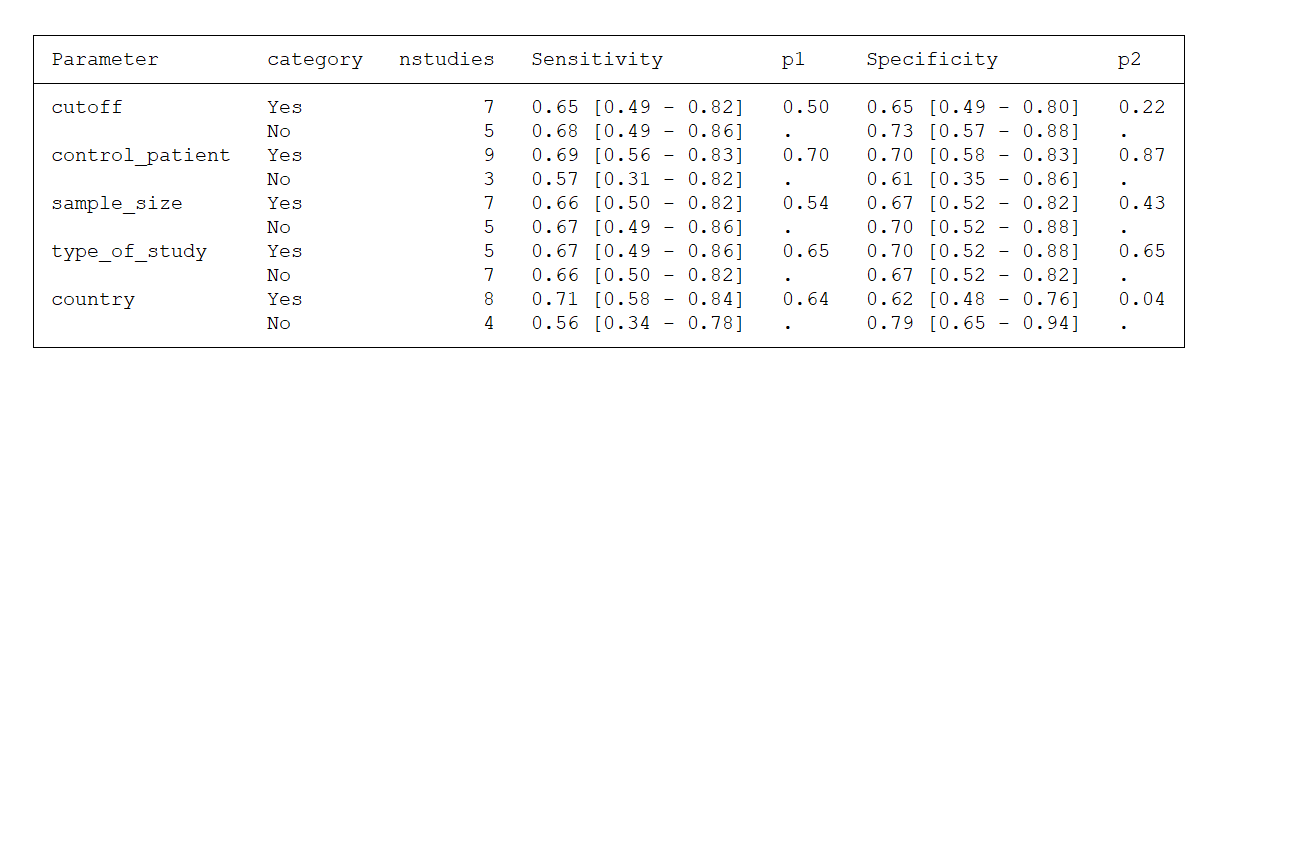

Supplement: Supplementary file 7 — Additional file 7: Suppl. Fig. 6. Summary of meta-regression analysis. [file 12879_2021_6150_MOESM7_ESM.tif]
